# Natural Experiments: An Overview of Methods, Approaches, and Contributions to Public Health Intervention Research

**DOI:** 10.1146/annurev-publhealth-031816-044327

**Published:** 2017-01-11

**Authors:** Peter Craig, Srinivasa Vittal Katikireddi, Alastair Leyland, Frank Popham

**Affiliations:** MRC/CSO Social and Public Health Sciences Unit, University of Glasgow, Glasgow G2 3QB, United Kingdom

**Keywords:** population health interventions, evaluation methods, causal inference

## Abstract

Population health interventions are essential to reduce health inequalities and tackle other public health priorities, but they are not always amenable to experimental manipulation. Natural experiment (NE) approaches are attracting growing interest as a way of providing evidence in such circumstances. One key challenge in evaluating NEs is selective exposure to the intervention. Studies should be based on a clear theoretical understanding of the processes that determine exposure. Even if the observed effects are large and rapidly follow implementation, confidence in attributing these effects to the intervention can be improved by carefully considering alternative explanations. Causal inference can be strengthened by including additional design features alongside the principal method of effect estimation. NE studies often rely on existing (including routinely collected) data. Investment in such data sources and the infrastructure for linking exposure and outcome data is essential if the potential for such studies to inform decision making is to be realized.

## Introduction

Natural experiments (NEs) have a long history in public health research, stretching back to John Snow’s classic study of London’s cholera epidemics in the mid-nineteenth century. Since the 1950s, when the first clinical trials were conducted, investigators have emphasized randomized controlled trials (RCTs) as the preferred way to evaluate health interventions. Recently, NEs and other alternatives to RCTs have attracted interest because they are seen as the key to evaluating large-scale population health interventions that are not amenable to experimental manipulation but are essential to reducing health inequalities and tackling emerging health problems such as the obesity epidemic (15, 27, 40, 68, 76).

We follow the UK Medical Research Council guidance in defining NEs broadly to include any event not under the control of a researcher that divides a population into exposed and unexposed groups (16). NE studies use this naturally occurring variation in exposure to identify the impact of the event on some outcome of interest. Our focus here is on public health and other policy interventions that seek to improve population health or which may have important health impacts as a by-product of other policy goals. One key evaluation challenge is selective exposure to the intervention, leading exposed individuals or groups to differ from unexposed individuals or groups in characteristics associated with better or worse outcomes. Understanding and modeling the process(es) determining exposure to the intervention are therefore central to the design and conduct of NE studies.

Some authors define NEs more narrowly to include only those in which the process that determines exposure (often referred to as the assignment or data-generating process) is random or as-if random (22, pp. 15–16). Truly random assignment, although not unknown (14), is extremely rare in policy and practice settings. As-if randomness lacks a precise definition, and the methods proposed to identify as-if random processes (such as a good understanding of the assignment process and checks on the balance of covariates between exposed and unexposed groups) are those used to assess threats to validity in any study that attempts to make causal inferences from observational data. In the absence of a clear dividing line, we prefer to adopt a more inclusive definition and to assess the plausibility of causal inference on a case-by-case basis.

In the next section, we set out a general framework for making causal inferences in experimental and observational studies. The following section discusses the main approaches used NE studies to estimate the impact of public health interventions and to address threats to the validity of causal inferences. We conclude with brief proposals for improving the future use of NEs.

## Causal Inference in Trials and Observational Studies

The potential outcomes model provides a useful framework for clarifying similarities and differences between true experiments on the one hand and observational studies (including NEs) on the other hand (51). Potential outcomes refer to the outcomes that would occur if a person (or some other unit) were exposed simultaneously to an intervention and a control condition. As only one of those outcomes can be observed, causal effects must be inferred from a comparison of average outcomes among units assigned to an intervention or to a control group. If assignment is random, the groups are said to be exchangeable and the intervention’s average causal effect can be estimated from the difference in the average outcomes for the two groups. In a well-conducted RCT, randomization ensures exchangeability. In an observational study, knowledge of the assignment mechanism can be used to make the groups conditionally exchangeable, for example, by controlling for variables that influence both assignment and outcomes to the extent that these variables are known and accurately measured (34).

As well as showing why a control group is needed, this framework indicates why an understanding of the assignment process is so important to the design of an NE study. The methods discussed in the next section can be seen as different ways of achieving conditional exchangeability. The framework also usefully highlights the need to be clear about the kind of causal effect being estimated and, in particular, whether it applies to the whole population (such as an increase in alcohol excise duty) or a particular subset (such as a change in the minimum legal age for purchasing alcohol). A comparison of outcomes between groups assigned to the intervention or control condition provides an estimate of the effect of assignment, known as the intention-to-treat (ITT) effect, rather than the effect of the intervention itself. The two are necessarily the same only if there is perfect compliance. Some methods, such as fuzzy regression discontinuity (RD) and instrumental variables (IVs), estimate a different effect, the complier average causal effect (CACE), which is the effect of the intervention on those who comply with their allocation into the control or intervention group (11). Under certain assumptions, the CACE is equivalent to the ITT effect divided by the proportion of compliers. Which effect is relevant will depend on the substantive questions the study is asking. If the effect of interest is the ITT effect, as in a pragmatic effectiveness trial or a policy evaluation in which decision makers wish to know about the effect across the whole population, methods that estimate a more restricted effect may be less useful (20).

A related issue concerns extrapolation—using results derived from one population to draw conclusions about another. In a trial, all units have a known probability of being assigned to the intervention or control group. In an observational study, where exchangeability may be achieved by a method such as matching or by conditioning on covariates, intervention groups may be created whose members in practice have no chance of receiving the treatment (55). The meaning of treatment effects estimated in this way is unclear. Extrapolation may also be a problem for some NE studies, such as those using RD designs, which estimate treatment effects at a particular value of a variable used to determine assignment. Effects of this kind, known as local average treatment effects, may be relevant to the substantive concerns of the study, but researchers should bear in mind how widely results can be extrapolated, given the nature of the effects being estimated.

Table 1 summarizes similarities and contrasts between RCTs, NEs, and nonexperimental observational studies.

## Methods for Evaluating Natural Experiments

Key considerations when choosing an NE evaluation method are the source of variation in exposure and the size and nature of the expected effects. The source of variation in exposure may be quite simple, such as an implementation date, or quite subtle, such as a score on an eligibility test. Interventions that are introduced abruptly, that affect large populations, and that are implemented where it is difficult for individuals to manipulate their treatment status are more straightforward to evaluate. Likewise, effects that are large and follow rapidly after implementation are more readily detectable than more subtle or delayed effects. One example of the former is a study that assessed the impact of a complete ban in 1995 on the import of pesticides commonly used in suicide in Sri Lanka (32). Suicide rates had risen rapidly since the mid-1970s, then leveled off following a partial ban on pesticide imports in the early 1980s. After the complete ban, rates of suicide by self-poisoning fell by 50%. The decrease was specific to Sri Lanka, was barely offset by an increase in suicide by other methods, and could not be explained by changes in death recording or by wider socioeconomic or political trends.

Although NE studies are not restricted to interventions with rapid, large effects, more complicated research designs may be needed where effects are smaller or more gradual. Table 2 summarizes approaches to evaluating NEs. It includes both well-established and widely used methods such as difference-in-differences (DiD) and interrupted time series (ITS), as well as more novel approaches such as synthetic controls. Below, we describe these methods in turn, drawing attention to their strengths and limitations and providing examples of their use.

### Regression Adjustment

Standard multivariable models, which control for observed differences between intervention and control groups, can be used to evaluate NEs when no important differences in unmeasured characteristics between intervention and control groups are expected (see Model 1 in Appendix 1). Goodman et al. used data from the UK Millennium Cohort Study to evaluate the impact of a school-based cycle training scheme on children’s cycling behavior (29). The timing of survey fieldwork meant that some interviews took place before and others after the children received training. Poisson models were used to estimate the effect of training on cycling behaviors, with adjustment for a wide range of potential confounders. Previous evaluations that compared children from participating and nonparticipating schools found substantial effects on cycling behavior. In contrast, this study found no difference, suggesting that the earlier findings reflected the selective provision of training. The key strength of the study by Goodman et al. is the way the timing of data gathering in relation to exposure created well-balanced intervention and control groups. Without this overlap between data gathering and exposure to the intervention, there was a significant risk that unobserved differences between the groups would bias the estimates, despite adjusting for a wide range of observed confounders.

### Propensity Score–Based Methods

In a well-conducted RCT, random allocation ensures that intervention and control arms are balanced in terms of both measured and unmeasured covariates. In the absence of random allocation, the propensity score attempts to recreate the allocation mechanism, defined as the conditional probability of an individual being in the intervention group, given a number of covariates (65).

The propensity score is typically estimated using logistic regression, based on a large number of covariates, although alternative estimation methods are available. There are four principal ways to use the propensity score to obtain an estimated treatment effect: matching, stratification, inverse probability weighting, and covariate adjustment (7). Each method will adjust for differences in characteristics of the intervention and control groups and, in so doing, minimize the effects of confounding. The propensity score, however, is constrained by the covariates available and the extent to which they can collectively mimic the allocation to intervention and control groups.

Understanding the mechanism underlying allocation to intervention and control groups is key when deriving the propensity score. Sure Start Local Programmes (SSLPs), area-based interventions designed to improve the health and well-being of young children in England, were an example where, on an ITT basis, exposure to the intervention was determined by area of residence and would apply to everyone living in the area regardless of individual characteristics. Melhuish et al. (54) therefore constructed a propensity score at the area level, based on 85 variables, to account for differences between areas with and without SSLPs. Analysis was undertaken on individuals clustered within areas, stratified by the propensity of an area to receive the SSLP. The most deprived areas were excluded from the analysis because there were insufficient comparison areas.

Advantages of using the propensity score over simple regression adjustment include the complexity of the propensity score that can be created (through, for example, including higher-order terms and interactions), the ease of checking the adequacy of the propensity score as opposed to checking the adequacy of a regression model, and the ability to examine the extent to which intervention and control groups overlap in key covariates (7, 18), and thereby avoid extrapolation. Although in statistical terms the use of propensity scores may produce results that differ little from those obtained through traditional regression adjustment (70), they encourage clearer thinking about study design and particularly the assignment mechanism (66). When membership of the treatment and control groups varies over time, inverse probability weighting can be used to account for time-varying confounding (34), as in the study by Pega et al. (59) of the cumulative impact of tax credits on self-rated health.

### Difference-in-Differences

In its simplest form, the DiD approach compares change in an outcome among people who are newly exposed to an intervention with change among those who remain unexposed. Although these differences could be calculated from a 2 × 2 table of outcomes for each group at each time point, the effect is more usefully estimated from a regression with terms for group, period, and group-by-period interaction. The coefficient of the interaction term is the DiD estimator (Model 2 in Appendix 1).

DiD’s strength is that it controls for unobserved as well as observed differences in the fixed (i.e., time-invariant) characteristics of the groups and is therefore less prone to omitted variable bias caused by unmeasured confounders or measurement error. The method relies on the assumption that, in the absence of the intervention, preimplementation trends would continue. This common trends assumption may be violated by differential changes in the composition of the intervention or control groups or by other events (such as the introduction of another intervention) that affect one group but not the other. With data for multiple preimplementation time points, the common trends assumption can be investigated directly, and it can be relaxed by extending the model to include terms for group-specific trends. With more groups and time points, the risk that other factors may influence outcomes increases, but additional terms can be included to take account of time-varying characteristics of the groups.

De Angelo & Hansen (19) used a DiD approach to estimate the effectiveness of traffic policing in reducing road traffic injuries and fatalities by taking advantage of a NE provided by the state of Oregon’s failure to agree on a budget in 2003, which led to the layoff of more than one-third of Oregon’s traffic police force. A comparison of injury and fatality rates in Oregon with rates in two neighboring states before and after the layoff indicated that, after allowing for other factors associated with road traffic incidents, such as the weather and the number of young drivers, less policing led to a 12–14% increase in fatalities. Whereas De Angelo & Hansen’s study focused on an intervention in a single area, Nandi and colleagues (58) applied DiD methods to estimate the impact of paid maternity leave across a sample of 20 low- and middle-income countries.

DiD methods are not limited to area-based interventions. Dusheiko et al. (23) used the withdrawal of a financial incentive scheme for family doctors in the English National Health Service to identify whether it led to treatment rationing. Recent developments, such as the use of propensity scores, rather than traditional covariate adjustment, to account for group-specific time-varying characteristics, add additional complexity, but combining DiD with other approaches in this way may further strengthen causal inference.

### Interrupted Time Series

Alongside DiD, ITS methods are among the most widely applied approaches to evaluating NEs. An ITS consists of a sequence of count or continuous data at evenly spaced intervals over time, with one or more well-defined change points that correspond to the introduction of an intervention (69). There are many approaches to analyzing time series data (44). A straightforward approach is to use a segmented regression model, which provides an estimate of changes in the level and trend of the outcome associated with the intervention, controlling for preintervention level and trend (43, 75). Such models can be estimated by fitting a linear regression model, including a continuous variable for time since the start of the observation period, a dummy variable for time period (i.e., before/after intervention), and a continuous variable for time postintervention (Model 3 in Appendix 1). The coefficients of these variables measure the preintervention trend, the change in the level of the outcome immediately postintervention, and the change in the trend postintervention. Additional variables can be added to identify the effects of interventions introduced at other time points or to control for changes in level or trend of the outcome due to other factors. Lags in the effect of the intervention can be accounted for by omitting outcome values that occur during the lag period or by modeling the lag period as a separate segment (75). Successive observations in a time series are often related to one another, a problem known as serial autocorrelation. Unless autocorrelation is addressed, the standard errors will be underestimated, but models that allow for autocorrelation can be fitted using standard statistical packages.

By accounting for preintervention trends, well-conducted ITS studies permit stronger causal inference than do cross-sectional or simple prepost designs, but they may be subject to confounding by cointerventions or changes in population composition. Controlled ITS designs, which compare trends in exposed and unexposed groups or in outcomes that are not expected to change as a result of the intervention, can be used to strengthen causal inference still further; in addition, standardization can be used to control for changes in population composition. A common shortcoming in ITS analyses is a lack of statistical power (61). Researchers have published a range of recommendations for the number of data points required, but statistical power also depends on the expected effect size and the degree of autocorrelation. Studies with few data points will be underpowered unless the effect size is large. Zhang et al. (79) and Mcleod & Vingilis (53) provide methods for calculating statistical power for ITS studies.

Robinson et al. (64) applied controlled ITS methods to commercially available alcohol sales data to estimate the impact of a ban on the offer of multipurchase discounts by retailers in Scotland. Because alcohol sales vary seasonally, the researchers fitted models that took account of seasonal autocorrelation, as well as trends in sales in England and Wales where the legislation did not apply. After adjusting for sales in England and Wales, the study found a 2% decrease in overall sales, compared with a previous study’s finding of no impact using DiD methods applied to self-reported alcohol purchase data.

### Synthetic Controls

The difficulty of finding control areas that closely match the background trends and characteristics of the intervention area is a significant challenge in many NE studies. One solution is to use a synthetic combination of areas rather than the areas themselves as controls. Methods for deriving synthetic controls and using them to estimate the impact of state-, region-, or national-level policies were developed by political scientists (1–4) and are now being applied to many health and social policies (8, 9, 17, 30, 45, 62, 67).

A synthetic control is a weighted average of control areas that provides the best visual and statistical match to the intervention area on the preintervention values of the outcome variable and of predictors of the outcome. Although the weights are based on observed characteristics, matching on the outcome in the preintervention period minimizes differences in unobserved fixed and time-varying characteristics. The difference between the postintervention trend in the intervention and synthetic control provides the effect estimate. Software to implement the method is available in a number of statistical packages (2).

Abadie et al. (1) used synthetic controls to evaluate a tobacco control program introduced in California in 1988, which increased tobacco taxes and earmarked the revenues for other tobacco control measures. The comparator was derived from a donor pool of other US states, excluding any states that had implemented extensive tobacco control interventions. A weighted combination of five states, based on pre-1988 trends in cigarette consumption and potential confounders, formed the synthetic control. Comparison of the postintervention trends in the real and synthetic California suggested a marked reduction in tobacco consumption as a result of the program.

The synthetic control method can be seen as an extension of the DiD method, with a number of advantages. In particular, it relaxes the requirement for a geographical control that satisfies the parallel trends assumption and relies less on subjective choices of control areas. A practical limitation, albeit one that prevents extrapolation, is that if the intervention area is an outlier, for example if California’s smoking rate in 1988 was higher than those of all other US states, then no combination of areas in the donor pool can provide an adequate match. Another limitation is that conventional methods of statistical inference cannot be applied, although Abadie et al. (1) suggest an alternative that compares the estimated effect for the intervention area with the distribution of placebo effects derived by comparing each area in the donor pool with its own synthetic control.

### Instrumental Variables

**Figure 1:**
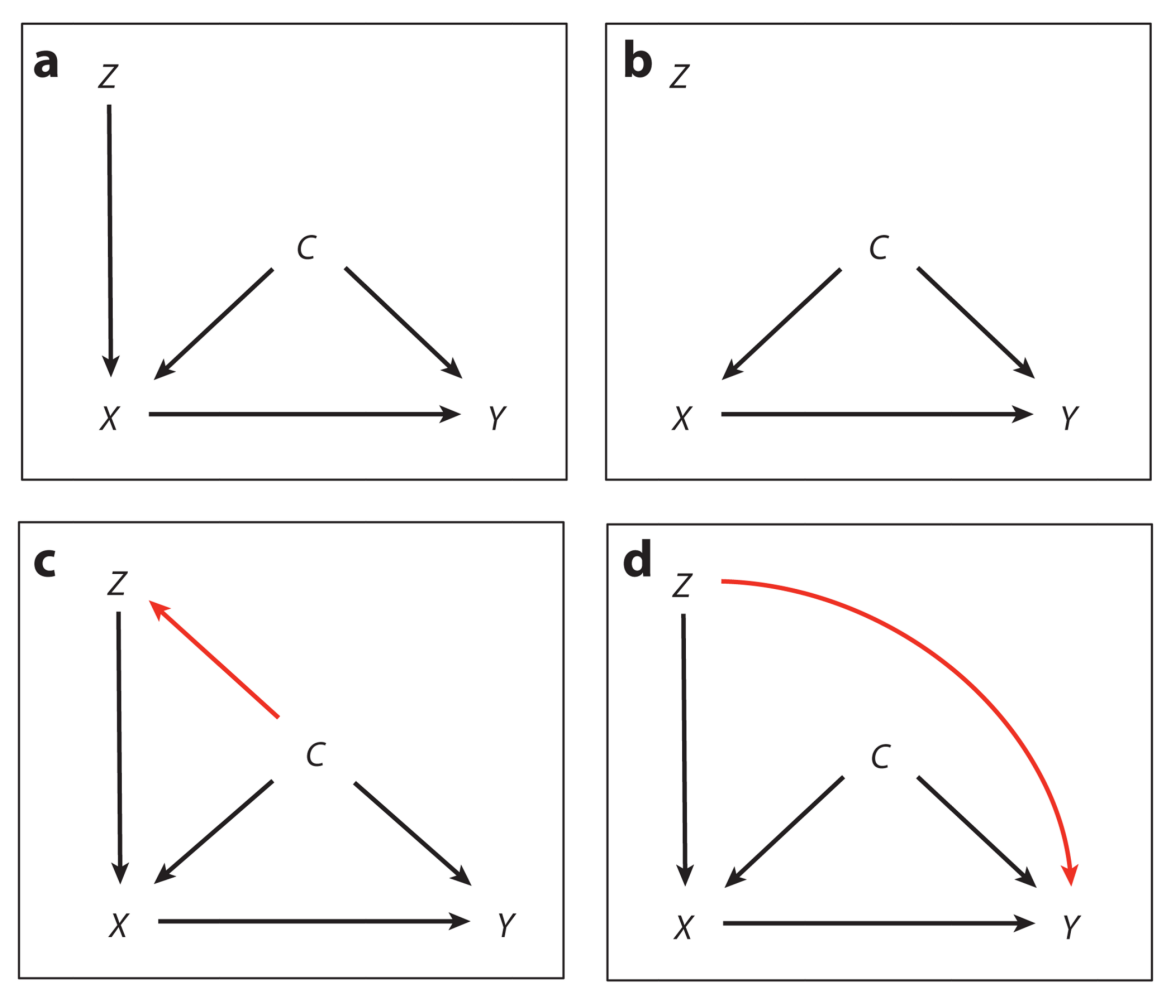
Directed acyclic graphs illustrating the assumptions of instrumental variable (IV) analysis. (*a*) The variable *Z* is associated with outcome *Y* only through its association with exposure *X*, so it can be considered a valid instrument of *X*. (*b*) *Z* is not a valid instrument owing to a lack of any association with outcome *Y*. (*c*) *Z* is not a valid instrument owing to its association with confounder *C*. (*d*) *Z* is not a valid instrument owing to its direct association with *Y*.

IV methods address selective exposure to an intervention by replacing a confounded direct measure of exposure with an unconfounded proxy measure, akin to treatment assignment in an RCT (33). To work in this way, an IV must be associated with exposure to the intervention, must have no association with any other factors associated with exposure, and must be associated with outcomes only through its association with exposure to the intervention (Figure 1).

IVs that satisfy the three conditions offer a potentially valuable solution to the problem of unobserved as well as observed confounders. Estimating an intervention’s effect using IVs can be viewed as a two-stage process (Models 5.1 and 5.2 in Appendix 1). In the first stage, a prediction of treatment assignment is obtained from a regression of the treatment variable on the instruments. Fitted values from this model replace the treatment variable in the outcome regression (41).

IVs are widely used in econometric program evaluation and have attracted much recent interest in epidemiology, particularly in the context of Mendelian randomization studies, which use genetic variants as instruments for environmental exposures (25, 36, 48). IV methods have not yet been widely used to evaluate public health interventions because it can be difficult to find suitable instruments and to demonstrate convincingly, using theory or data, that they meet the second and third conditions above (35, 71). A recent example is the study by Ichida et al. (39) of the effect of community centers on improving social participation among older people in Japan, using distance to the nearest center as an instrument for intervention receipt. Another study, by Yen et al. (78), considers the effect of food stamps on food insecurity, using a range of instruments, including aspects of program administration that might encourage or discourage participation in the food stamp program. Given the potential value of IVs, as one of a limited range of approaches for mitigating the problems associated with unobserved confounders, and their widespread use in related fields, they should be kept in mind should opportunities arise (35).

### Regression Discontinuity

Age, income, and other continuous variables are often used to determine entitlement to social programs, such as means-tested welfare benefits. The RD design uses such assignment rules to estimate program impacts. RD is based on the insight that units with values of the assignment variable just above or below the cutoff for entitlement will be similar in other respects, especially if there is random error in the assignment variable (11). This similarity allows the effect of the program to be estimated from a regression of the outcome on the assignment variable (often referred to as the running or forcing variable) and a dummy variable denoting exposure (treatment), with the coefficient of the dummy identifying the treatment effect (Model 4 in Appendix 1). Additional terms are usually included in the model to allow slopes to vary above and below the cutoff, allow for nonlinearities in the relationship between the assignment and outcome variables, and deal with residual confounding.

**Figure 2:**
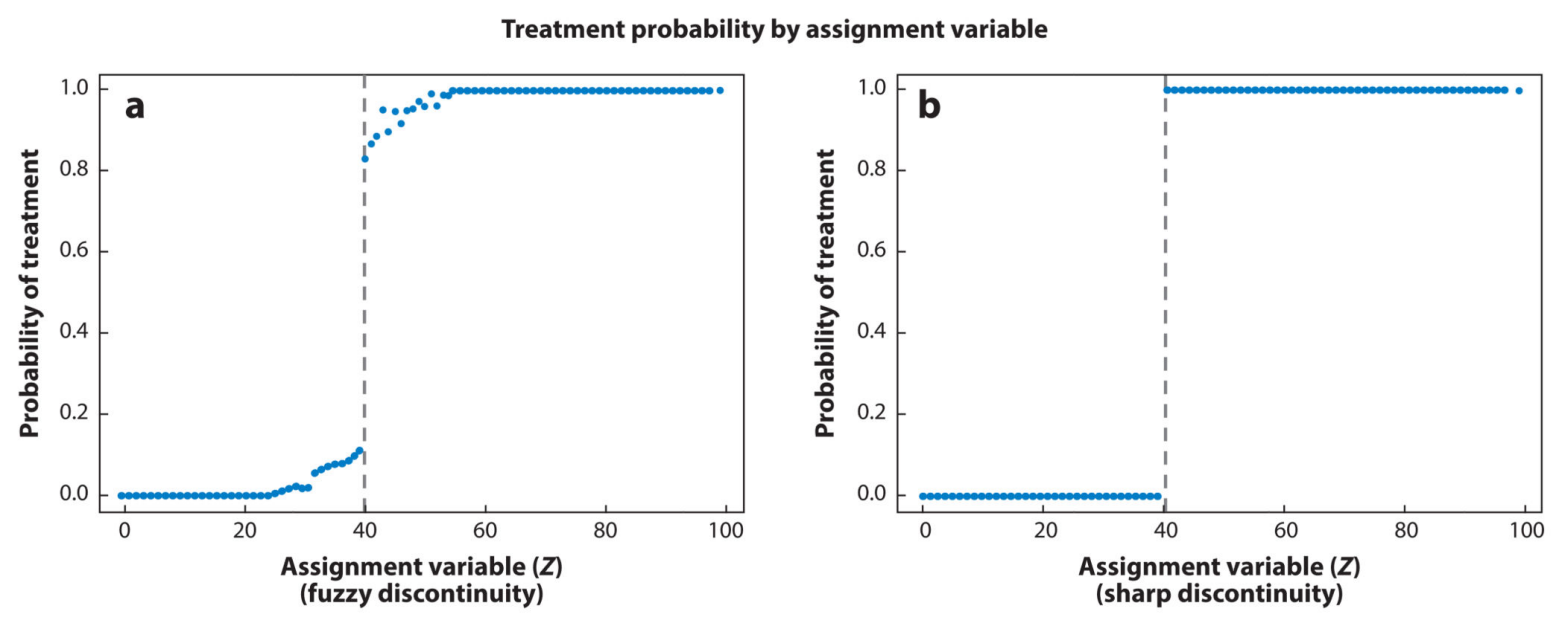
Probability of receiving treatment in fuzzy and sharp regression discontinuity designs. (*a*) A fuzzy regression discontinuity: probability of treatment changes gradually at values of the assignment variable close to the cutoff. (*b*) A sharp regression discontinuity: probability of treatment changes from 0 to 1 at the cutoff. Source: Reproduced from Moscoe (2015) (57) with permission from Elsevier.

Visual checks play an important role in RD studies. Plots of treatment probability (Figure 2) and outcomes against the assignment variable can be used to identify discontinuities that indicate a treatment effect, and a histogram of the assignment variable can be plotted to identify bunching around the cutoff that would indicate manipulation of treatment assignment. Scatterplots of covariates against assignment can be used to check for continuity at the cutoff that would indicate whether units above and below the cutoff are indeed similar (57).

The RD estimator need not be interpreted only as the effect of a unit’s exposure to the program (treatment) right at the cutoff value (47), but the assumption that units above and below the cutoff are similar except in their exposure to the program becomes less tenable as distance from the cutoff increases. Usual practice is to fit local linear regressions for observations within a narrow band on either side of the cutoff. Restricting the analysis in this way also means that nonlinearities in the relationship between the forcing and outcome variables are less important. One drawback is that smaller numbers of observations will yield less precise estimates, so the choice involves a trade-off between bias and precision.

The above approach works when the probability of a treatment jumps from 0 to 1 at the cutoff, which is known as sharp RD (Figure 2b). If exposure is influenced by factors other than the value of the forcing variable, for example because administrators can exercise discretion over whom to include in the program or because individuals can, to some extent, manipulate their own assignment, the probability of treatment may take intermediate values close to the cutoff (Figure 1b) and a modified approach known as fuzzy RD should be applied. This process uses the same two-stage approach to estimation as does an IV analysis (Models 5.1 and 5.2 in Appendix 1).

One example of a sharp RD design is Ludwig & Miller’s (52) analysis of the US Head Start program. Help with applications for Head Start funding was targeted to counties with poverty rates of 59% or greater. This targeting led to a lasting imbalance in the receipt of Head Start funds among counties with poverty rates above and below the cutoff. Ludwig & Miller used local linear regressions of mortality on poverty rates for counties with poverty rates between 49% and 69%; the impact of the Head Start funding was defined as the difference between the estimated mortality rates at the upper and lower limits of this range. They found substantial reductions in mortality from causes amenable to Head Start but not from other causes of death or in children whose ages meant they were unlikely to benefit from the program.

Andalon (6) used a fuzzy RD design to investigate the impact of a conditional cash transfer program on obesity and overweight. Mexico’s *Opportunidades* program provided substantial cash subsidies to households in rural communities that scored below a poverty threshold, which were conditional on school and health clinic attendance. There were a range of other ad hoc adjustments to eligibility criteria, creating a fuzzy rather than a sharp discontinuity in participation at the poverty cutoff. Andalon used two-stage least squares regression to estimate the effect of eligibility (based on the poverty score) on program participation and the effect of predicted participation on obesity and overweight. The author found no effect for men but a substantial reduction in obesity among women. Further testing indicated no bunching of poverty scores around the cutoff and no significant discontinuity at the cutoff in a range of covariates. Inclusion of the covariates in the outcome regressions had little effect on the estimates, further supporting the assumption of local randomization.

RD methods are widely regarded as the closest approximation of an observational study to an RCT (5), but their real value derives from their wide applicability to the evaluation of social programs for which eligibility is determined by a score on some form of continuous scale and also from their reliance on relatively weak, directly testable assumptions. One key shortcoming is that restricting the bandwidth to reduce bias results in a loss of precision (46, 73), and estimates that may hold over only a small segment of the whole population exposed to the intervention. This restriction to a subset of the population may not matter if the intervention is expected to affect outcomes locally, as in the case of a minimum legal drinking age or if the substantive focus of the study is on the effect of a small change in the assignment rule. It is more serious when the outcome of interest is the effect on the whole population.

## Strengthening Inference in Natural Experimental Studies

Causal inference can be strengthened in NE studies by the inclusion of additional design features alongside the principal method of effect estimation. Studies should be based on a clear theoretical understanding of how the intervention achieves its effects and the processes that determine exposure. Even if the observed effects are large and rapidly follow implementation, confidence in attributing them to the intervention can be markedly improved by a detailed consideration of alternative explanations.

Qualitative research can strengthen the design of RCTs of complex public health interventions (10, 56), and this argument applies equally to NEs (38). Qualitative research undertaken in preparation for, or alongside, NE studies can help to identify which outcomes might change as a consequence of the intervention and which are priorities for decision makers (42). It can also improve understanding of the processes that determine exposure, factors associated with intervention delivery and compliance, mechanisms by which outcomes are realized, and the strengths and limitations of routinely collected measures of exposures and outcomes (13). Qualitative studies conducted alongside the quantitative evaluation of Scotland’s smoke-free legislation have been used to assess compliance with the intervention (24) and to identify a range of secondary outcomes such as changes in smoking behavior within the home (60). Qualitative methods for identifying the effects of interventions have also been proposed, but further studies are needed to establish their validity and usefulness (63, 72, 77).

Quantitative methods for strengthening inference include the use of multiple estimation methods within studies, replication studies, and falsification tests. Tests specific to particular methods, such as visual checks for discontinuities in RD and ITS studies, can also be used. Good-quality NE studies typically use a range of approaches. Comparing results obtained using different methods can be used to assess the dependence of findings on particular assumptions (50). Such comparisons are particularly useful in early applications of novel methods whose strengths and weaknesses are not fully understood (49).

Falsification or placebo tests assess the plausibility of causal attribution by checking for the specificity of effects. One such approach is to use nonequivalent dependent variables to measure changes in outcomes that are not expected to respond to the intervention. They serve as indicators of residual confounding or the effects of other interventions introduced alongside the study intervention. A related approach is to use false implementation dates and to compare changes associated with those dates with effects estimated for the real implementation date. A similar test used in synthetic control studies involves generating placebo effects by replacing the intervention area with each of the areas in the donor pool in turn and then comparing the estimated intervention effect with the distribution of placebo effects (1, 2).

Most NE studies are conducted retrospectively, using data collected before the study is planned. Ideally, an analysis protocol, setting out hypotheses and methods, should be developed before any data analysis is conducted (21). Even when such protocols are published, they do not provide a perfect safeguard against selective reporting of positive findings. Replication studies, which by definition retest a previously published hypothesis, are a valuable additional safeguard against retrospectively fitting hypotheses to known features of the data. Reporting of NE studies of all kinds may also be improved by following established reporting guidelines such as STROBE (Strengthening the Reporting of Observational Studies in Epidemiology) (74) or TREND (Transparent Reporting of Evaluations with Nonrandomized Designs) (28).

## Conclusions

NE approaches to evaluation have become topical because they address researchers’ and policy makers’ interests in understanding the impact of large-scale population health interventions that, for practical, ethical, or political reasons, cannot be manipulated experimentally. We have suggested a pragmatic approach to NEs. NEs are not the answer to every evaluation question, and it is not always possible to conduct a good NE study whenever an RCT would be impractical. Choices among evaluation approaches are best made according to specific features of the intervention in question, such as the allocation process, the size of the population exposed, the availability of suitable comparators, and the nature of the expected impacts, rather than on the basis of general rules about which methods are strongest, regardless of circumstances. Availability of data also constrains the choice of methods. Where data allow, combining methods and comparing results are good ways to avoid overdependence on particular assumptions. Having a clear theory of change based on a sound qualitative understanding of the causal mechanisms at work is just as important as sophisticated analytical methods.

Many of the examples discussed above use routinely collected data on outcomes such as mortality, road traffic accidents, and hospital admissions and data on exposures such as poverty rates, alcohol sales, and tobacco consumption. Continued investment in such data sources, and in population health surveys, is essential if the potential for NEs to contribute to the evidence base for policy making is to be realized. Recent investments in infrastructure to link data across policy sectors for research purposes are a welcome move that should increase opportunities to evaluate NEs (12, 26, 37). Funding calls for population health research proposals should take a similarly even-handed approach to specifying which approaches would be acceptable and should emphasize the importance of developing a clear theory of change, carefully testing assumptions, and comparing estimates from alternative methods.

## Supplementary Material

Appendix

## Figures and Tables

**Table 1:** Similarities and differences between RCTs, NEs, and observational studies

Type of study	Is the intervention well defined?	How is the intervention assigned?	Does the design eliminate confounding?	Do all units have a nonzero chance of receiving the treatment?
RCTs	A well-designed trial should have a clearly defined intervention described in the study protocol.	Assignment is under the control of the research team; units are randomly allocated to intervention and control groups.	Randomization means that, in expectation, there is no confounding, but imbalances in covariates could arise by chance.	Randomization means that every unit has a known chance of receiving the treatment or control condition.
NEs	Natural experiments are defined by a clearly identified intervention, although details of compliance, dose received, etc., may be unclear.	Assignment is not under the control of the research team; knowledge of the assignment process enables confounding due to selective exposure to be addressed.	Confounding is likely due to selective exposure to the intervention and must be addressed by a combination of design and analysis.	Possibility of exposure may be unclear and should be checked. For example, RD designs rely on extrapolation but assume that at the discontinuity units could receive either treatment or no treatment.
Nonexperimental observational studies	There is usually no clearly defined intervention, but there may be a hypothetical intervention underlying the comparison of exposure levels.	There is usually no clearly defined intervention and there may be the potential for reverse causation (i.e., the health outcome may be a cause of the exposure being studied) as well as confounding.	Confounding is likely due to common causes of exposure and outcomes and can be addressed, in part, by statistical adjustment; residual confounding is likely, however.	Possibility of exposure is rarely considered in observational studies so there is a risk of extrapolation unless explicitly addressed.

Abbreviations: NE, natural experiment; RCT, randomized controlled trial; RD, regression discontinuity.

**Table 2:** Approaches to evaluating NEs

Description	Advantages/disadvantages	Examples
**Prepost**		
Outcomes of interest compared in a population pre- and postexposure to the intervention	Requires data in only a single population whose members serve as their own controls Assumes that outcomes change only as a result of exposure to the intervention	Effect of pesticide import bans and suicide in Sri Lanka (32)
**Regression adjustment**		
Outcomes compared in exposed and unexposed units, and a statistical model fitted to take account of differences between the groups in characteristics thought to be associated with variation in outcomes	Takes account of factors that may cause both the exposure and the outcome Assumes that all such factors have been measured accurately so that there are no unmeasured confounders	Effect of repeal of handgun laws on firearm-related murders in Missouri (17) Effect of a cycle training scheme on cycling rates in British schoolchildren (29)
**Propensity scores**		
Likelihood of exposure to the intervention calculated from a regression model and either used to match exposed and unexposed units or fitted in a model to predict the outcome of interest	Allows balanced comparisons when many factors are associated with exposure Assumes that all such factors have been measured accurately so that there are no unmeasured confounders	Effect of the Sure Start scheme in England on the health and well-being of young children (54)
**Difference-in-differences**		
Change in the outcome of interest pre- and postintervention compared in exposed and unexposed groups	Uses differencing procedure to control for variation in both observed and unobserved fixed characteristics Assumes that there are no group-specific trends that may influence outcomes—the parallel trends assumption	Effect of traffic policing on road traffic accidents in Oregon (19) Effect of paid maternity leave on infant mortality in LMICs (58)
**Interrupted time series**		
Trend in the outcome of interest compared pre- and postintervention, using a model that accounts for serial correlation in the data and can identify changes associated with introduction of the intervention. Change also compared in exposed and unexposed populations in controlled time series analyses	Provides a powerful and flexible method for dealing with trend data Requires substantial numbers of pre- and postintervention data points; controlled time series analyses may not be possible if the trends in the intervention and control area differ markedly	Effect of a multibuy discount ban on alcohol sales in Scotland (64) Effect of 20-mph zones on road traffic casualties in London, UK (31)
**Synthetic controls**		
Trend in the outcome of interest compared in an intervention area and a synthetic control area, representing a weighted composite of real areas that mimics the preintervention trend	Does not rely on the parallel trends assumption or require identification of a closely matched geographical control May not be possible to derive a synthetic control if the intervention area is an outlier	Effect of a ban on the use of *trans*-fats on heart disease in Denmark (62) Effect of antitobacco laws on tobacco consumption in California (1)
**Regression discontinuity**		
Outcomes compared in units defined by scores just above and below a cutoff in a continuous forcing variable that determines exposure to an intervention	Units with scores close to the cutoff should be very similar to one another, especially if there is random error in the assignment variable; some key assumptions can be tested directly Estimates the effects for units with scores close to the cutoff, which may not be generalizable to units with much higher or lower scores on the forcing variable; there is a trade-off between statistical power (which requires including as many people as possible near the cutoff) and minimizing potential confounding (by including only those very close to the cutoff)	Effect of the Head Start program on child mortality in the United States (52) Effects of conditional cash transfers on rates of overweight/obesity in Mexico (6)
**Instrumental variables**		
A variable associated with exposure to the intervention, but not with other factors associated with the outcome of interest, used to model the effect of the intervention	An instrumental variable that satisfies these assumptions should provide an unconfounded estimate of the effect of the intervention Such variables are rare, and not all of the assumptions can be tested directly	Effect of food stamps on food insecurity (78) Effect of community salons on social participation and self-rated health among older people in Japan (39)

Abbreviations: LMIC, low- and middle-income countries; NE, natural experiment.
