# Supplementary material for: Natural Experiments: An Overview of Methods, Approaches, and Contributions to Public Health Intervention Research
Source: Annu Rev Public Health. Author manuscript; Available in PMC 2019 Apr 26. (PMC6485604; doi:10.1146/annurev-publhealth-031816-044327)
Supplement: Appendix [file NIHMS78383-supplement-Appendix.pdf]

APPENDIX 1: STATISTICAL MODELS FOR NATURAL EXPERIMENTAL STUDIES

The standard multivariate regression model can be used to estimate the effect of exposure to an intervention (E) on the outcome (Y), with adjustment for a measured confounder (X),

$$Y_i = \beta_0 + \beta_1 E_i + \beta_2 X_i + \varepsilon_i. \quad \text{(Model 1)}$$

Terms can be added to adjust for other confounders.

In a DiD analysis, observations are made on different units i at times t and the regression model includes an additional term for the period (P) in which the observation took place (coded 0 for preintervention or 1 for postintervention), and an interaction term between the period and exposure, which provides the effect estimate β_3:

$$Y_{it} = \beta_0 + \beta_1 E_i + \beta_2 P_t + \beta_3 E_i \times P_t + \varepsilon_{it}. \quad \text{(Model 2)}$$

Segmented regression models estimate the baseline level of the outcome, the trend in the outcome before the intervention, the change that occurs at the point when the intervention is introduced, and the trend postintervention:

$$Y_t = \beta_0 + \beta_1 U_t + \beta_2 P_t + \beta_3 V_t + \varepsilon_t, \quad \text{(Model 3)}$$

where U is the time from the start of the observation period, P is again a dummy variable indicating the preintervention ($P = 0$) and postintervention ($P = 1$) periods, and V is the time postintervention. Additional terms can be added to allow for multiple interventions, to model a lag period postintervention if it takes time for the effects to appear, or to account for serial correlation in the data.

In a sharp RD analysis, the model includes the forcing variable (Z), and the exposure variable (E) takes a value of 1 when Z is equal to or greater than the cutoff (C) that determines exposure and 0 otherwise. In Model 4, β_1 provides an estimate of the change in Y at the cutoff, and β_2 and β_3 estimate the slope below and above the cutoff. The analysis is usually restricted to values of Z close to C:

$$Y_i = \beta_0 + \beta_1 E_i + \beta_2 (1 - E_i)(Z_i - C) + \beta_3 E_i (Z_i - C) + \varepsilon_i. \quad \text{(Model 4)}$$

Models 1–4 estimate an average treatment effect across the whole exposed population. The two-stage least squares (2SLS) models used in fuzzy RD and IV studies estimate a complier average causal effect, i.e., the effect of the intervention on those who comply with their assignment. The first stage predicts the probability of exposure π, with C representing the cutoff and Z the forcing variable in an RD analysis and the IV and a confounding variable in an IV analysis:

$$E_i \sim \mathit{Bernoulli}(\pi_i) \quad \text{(Model 5.1)}$$

$$g(\pi_i) = \beta_0^* + \beta_1^* C_i + \beta_2^* Z_i,$$

where $g(.)$ denotes an appropriate link function, e.g., logit, probit.

The second stage uses the expected probabilities from the first stage to provide the effect estimate, β_1:

$$Y_i = \beta_0 + \beta_1 \hat{\pi}_i + \beta_2 Z_i + \varepsilon_i, \quad \text{(Model 5.2)}$$

where $\hat{\pi}_i$ is the predicted probability of exposure from Model 5.1, e.g., for a logit link,

$$\hat{\pi}_i = \frac{1}{1 + \exp\left\{-\left(\hat{\beta}_0^* + \hat{\beta}_1^* C_i + \hat{\beta}_2^* Z_i\right)\right\}}.$$

Note that Model 5.2 includes the forcing variable and any other confounders from Model 5.1.

Annu. Rev. Public Health 2017.38:39-56. Downloaded from www.annualreviews.org
Access provided by Glasgow University on 06/26/18. See copyright for approved use.
